# Effectiveness of a Mental Health Chatbot for People With Chronic Diseases: Randomized Controlled Trial

**DOI:** 10.2196/50025

**Published:** 2024-05-30

**Authors:** A Luke MacNeill, Shelley Doucet, Alison Luke

**Affiliations:** 1 Centre for Research in Integrated Care University of New Brunswick Saint John, NB Canada; 2 Department of Nursing and Health Sciences University of New Brunswick Saint John, NB Canada

**Keywords:** chatbot, chronic disease, arthritis, diabetes, mental health, depression, anxiety, stress, effectiveness, application

## Abstract

**Background:**

People with chronic diseases tend to experience more mental health issues than their peers without these health conditions. Mental health chatbots offer a potential source of mental health support for people with chronic diseases.

**Objective:**

The aim of this study was to determine whether a mental health chatbot can improve mental health in people with chronic diseases. We focused on 2 chronic diseases in particular: arthritis and diabetes.

**Methods:**

Individuals with arthritis or diabetes were recruited using various web-based methods. Participants were randomly assigned to 1 of 2 groups. Those in the treatment group used a mental health chatbot app (Wysa [Wysa Inc]) over a period of 4 weeks. Those in the control group received no intervention. Participants completed measures of depression (Patient Health Questionnaire–9), anxiety (Generalized Anxiety Disorder Scale–7), and stress (Perceived Stress Scale–10) at baseline, with follow-up testing 2 and 4 weeks later. Participants in the treatment group completed feedback questions on their experiences with the app at the final assessment point.

**Results:**

A total of 68 participants (n=47, 69% women; mean age 42.87, SD 11.27 years) were included in the analysis. Participants were divided evenly between the treatment and control groups. Those in the treatment group reported decreases in depression (*P*<.001) and anxiety (*P*<.001) severity over the study period. No such changes were found among participants in the control group. No changes in stress were reported by participants in either group. Participants with arthritis reported higher levels of depression (*P*=.004) and anxiety (*P*=.004) severity than participants with diabetes over the course of the study, as well as higher levels of stress (*P*=.01); otherwise, patterns of results were similar across these health conditions. In response to the feedback questions, participants in the treatment group said that they liked many of the functions and features of the app, the general design of the app, and the user experience. They also disliked some aspects of the app, with most of these reports focusing on the chatbot’s conversational abilities.

**Conclusions:**

The results of this study suggest that mental health chatbots can be an effective source of mental health support for people with chronic diseases such as arthritis and diabetes. Although cost-effective and accessible, these programs have limitations and may not be well suited for all individuals.

**Trial Registration:**

ClinicalTrials.gov NCT04620668; https://www.clinicaltrials.gov/study/NCT04620668

## Introduction

Chronic diseases affect billions of people around the world [1]. They have been identified as a leading cause of disability [2] and account for over 70% of global deaths [3]. Although the physical burden of chronic diseases is widely recognized, the link between chronic diseases and mental health is sometimes overlooked. Researchers have found that people with chronic diseases tend to report higher levels of depression and anxiety than their peers without these health conditions [4-7]. Furthermore, people with chronic diseases often experience more chronic disease symptoms and a worse medical prognosis when they have comorbid mental health issues [8-10]. These findings highlight the importance of mental health for people living with chronic diseases and suggest that mental health support could be a valuable resource for this population.

People with chronic diseases who are seeking mental health support may find some benefit in mental health chatbots. Mental health chatbots are software programs that are designed to engage in supportive, humanlike conversation with individuals [11,12]. They offer mental health guidance, coaching, and education with the aim of reducing negative mental health symptoms and improving mental well-being. These programs are not intended to replace real mental health professionals. However, they are an accessible and cost-effective alternative to conventional mental health services that could benefit some people. They may be particularly useful for people with chronic diseases, as these individuals often face barriers to accessing mental health treatment (financial barriers, limited mobility, etc) [13-17]. A growing body of research has shown that mental health chatbots can reduce symptoms of depression, anxiety, and stress in nonclinical populations [18-22]. Less is known about their effectiveness in patient populations, although some research indicates that they may be beneficial for people with musculoskeletal conditions [23] and chronic pain [24].

The purpose of this study was to determine whether a mental health chatbot can improve mental health in people with chronic diseases. This study focused on 2 chronic diseases in particular, namely arthritis and diabetes. Arthritis and diabetes are among the most common chronic diseases, each affecting hundreds of millions of people worldwide [1]. Both health conditions have been linked to elevated levels of negative mental health symptoms [4-6], suggesting that there is an unmet need for mental health support among people living with these conditions. Individuals with arthritis or diabetes were recruited for a 4-week study and randomly assigned to 1 of 2 groups. One group used the mental health app Wysa (Wysa Inc), which features a fully automated chatbot that supports mental health and well-being. The other group received no intervention. We hypothesized that participants who used the app would report greater decreases in levels of depression, anxiety, and stress than participants who received no intervention.

As a secondary purpose of this study, we sought to gain a better understanding of people’s experiences with the mental health app. Participants who used the app were asked to provide feedback on their experiences with the program, particularly what they liked and disliked about their interactions with it. This information was expected to offer some indirect insight into the usefulness and effectiveness of the app. It was also expected to inform future revisions of the app and provide guidance and direction to developers who intend to create similar programs in the future.

## Methods

### Design

This study was a randomized controlled trial conducted using an embedded mixed methods approach [25]. Web-based surveys were used to collect quantitative data on participants’ mental health and well-being, as well as qualitative feedback on users’ experiences with the intervention. The study was registered with ClinicalTrials.gov (ID NCT04620668) and reported in accordance with the CONSORT (Consolidated Standards of Reporting Trials)–EHEALTH checklist [26].

### Sample Size

The required sample size for the quantitative portion of the analysis was determined using G*Power (Heinrich-Heine-Universität) power analysis software. The power analysis indicated that 40 participants would be required to detect interactions between the various study groups, assuming standard α level (.05) and power (.80), a moderate effect size (η_p_^2^=.06), and a moderate correlation between repeated measures (.50). Moderate effect size and correlation values were used as default values due to the lack of previous research on the use of mental health chatbots with chronic disease populations. A sample size of 60 participants was targeted to account for attrition and provide a more robust sample for follow-up testing.

### Inclusion and Exclusion Criteria

This study included Canadian residents between the ages of 19 and 65 years who had a diagnosis of arthritis or diabetes. Individuals were excluded from participation if they were receiving ongoing treatment from a mental health professional, if they were already using a digital mental health program, or if they started or experienced a dosage change in a mental health medication within the previous month. In addition, individuals needed to have access to a smartphone with an active internet connection to participate.

### Participant Recruitment

Participants were recruited through social media channels (eg, Facebook), classified websites (eg, Kijiji), and websites of relevant organizations (eg, the Arthritis Society). Recruitment occurred from October 2021 to October 2022, with pauses during holiday periods and peak phases of the COVID-19 pandemic to help minimize the impact of these events on the results. Enrollment in the study occurred on a rolling basis.

### Intervention

The study intervention was an artificial intelligence–enabled mental health app called Wysa. Wysa features a fully automated chatbot that draws from evidence-based treatments and techniques (eg, cognitive behavioral therapy, dialectical behavioral therapy, and motivational interviewing) to reduce negative mental health symptoms and improve mental well-being. It helps users manage a variety of issues, including loneliness and grief, sleep problems, and low confidence and self-esteem. Content is designed in conjunction with professional psychologists and approved by an advisory board comprised of mental health professionals.

Users download the Wysa app onto their smartphone and interact with the chatbot as needed using a text-based conversational interface. User input involves a combination of free text and restricted text (ie, scripted) responses. In addition to standard conversations with the chatbot, the app includes a variety of self-care exercises, most of which are delivered through the chatbot. The app also offers weekly reports and regular check-ins and reminders. No personally identifiable information is required for app use.

This study used a modified version of the publicly available Wysa app. The study version of the app had some added content for people with chronic diseases (eg, managing pain and depression resulting from health issues). It also omitted 1 feature of the publicly available app, namely the ability to access a human therapist for support (for a fee). This feature was removed from the study version of the app to ensure that the evaluation covered only the automated aspects of the app.

### Measures

A demographic questionnaire collected information about participant age, gender, and race or ethnicity. Information on participants’ health conditions was also collected. Additional questions and measures are described in greater detail in the following subsections.

#### Patient Health Questionnaire–9

Depression was assessed with the Patient Health Questionnaire–9 (PHQ-9) [27]. Individuals completing the measure are asked to report how often they experienced various symptoms of depression over the past 2 weeks, with responses rated on a scale of 0 (not at all) to 3 (nearly every day). Ratings are summed over the 9 items to obtain a composite score of depression severity. Higher scores indicate higher levels of depression severity. The developers of the PHQ-9 provided the following guidelines for interpretation: scores of 0-4 suggest minimal depression, scores of 5-9 suggest mild depression, scores of 10-14 suggest moderate depression, scores of 15-19 suggest moderately severe depression, and scores of 20 or greater suggest severe depression [27]. Past research has shown that the PHQ-9 is reliable and valid across a variety of populations, including patient populations [28,29]. Items on the PHQ-9 demonstrated high internal consistency in our study, with Cronbach α ranging from 0.87 to 0.91.

#### Generalized Anxiety Disorder Scale–7

Anxiety was assessed with the Generalized Anxiety Disorder Scale–7 (GAD-7) [30]. Individuals completing the measure are asked to report how often they experienced various symptoms of anxiety over the past 2 weeks, with responses rated on a scale of 0 (not at all) to 3 (nearly every day). Ratings are summed over the 7 items to obtain a composite score of anxiety severity. Higher scores indicate higher levels of anxiety severity. The developers of the GAD-7 provided the following guidelines for interpretation: scores of 0-4 suggest minimal anxiety, scores of 5-9 suggest mild anxiety, scores of 10-14 suggest moderate anxiety, and scores of 15 or greater suggest severe anxiety [30]. Past research has shown that the GAD-7 is reliable and valid across a variety of populations, including patient populations [28,31]. Items on the GAD-7 demonstrated high internal consistency in our study, with Cronbach α ranging from 0.92 to 0.93.

#### Perceived Stress Scale–10

Stress was assessed with the Perceived Stress Scale–10 (PSS-10) [32]. Individuals completing the measure are asked to report how often they experienced various indicators of stress over the past month, with responses rated on a scale of 0 (never) to 4 (very often). Items that are positively worded are reverse scored, and ratings are summed over the 10 items to obtain a composite score of perceived stress. Higher scores indicate higher levels of stress. The developers of the PSS-10 did not provide cutoffs for interpretation, although other researchers have used the following guidelines: scores of 0-13 suggest low stress, scores of 14-26 suggest moderate stress, and scores of 27-40 suggest high stress [33-36]. Past research has shown that the PSS-10 is reliable and valid across a variety of populations, including patient populations [37,38]. Items on the PSS-10 demonstrated high internal consistency in our study, with Cronbach α ranging from 0.87 to 0.91.

#### Feedback Questions

Two feedback questions were designed specifically for this study. These questions were open-ended (free response) questions asking users about their experiences with the Wysa app. More specifically, users were asked (1) “What did you like about interacting with the Wysa app? Please list as many things as possible” and (2) “What did you dislike about interacting with the Wysa app? Please list as many things as possible.”

### Procedure

The primary investigator contacted volunteers over the phone to assess the eligibility criteria, enroll eligible individuals in the study, and provide the study instructions. Upon enrollment, participants were randomly assigned to either a treatment group or a control group based on a randomization list created using the website RANDOM.ORG. The investigator was not blinded to group assignment, as participants required different study instructions during the phone call depending on their assignment. Participants assigned to the treatment group were asked to use the Wysa app over a period of 4 weeks. They were encouraged to interact with the chatbot at least twice per week, although they were not required to do so and were included in the analysis regardless of their usage. Participants assigned to the control group were not told about the intervention and were only aware that the study involved chronic diseases and mental health. Regardless of their group assignment, participants were informed during the call that they would need to complete questionnaires at certain points throughout the study (details in the next paragraph). At the end of the call, the primary investigator answered any questions that participants had about the study procedure.

Three rounds of questionnaires were administered to participants during the study. The questionnaires were hosted on the Qualtrics (Silver Lake) survey platform; links to the questionnaires were distributed through email. At baseline, participants completed an informed consent form; the demographic questionnaire; and the measures of depression (PHQ-9), anxiety (GAD-7), and stress (PSS-10). Participants in the treatment group also received instructions on how to download the app. Two weeks after baseline, participants completed the measures of depression, anxiety, and stress a second time. Four weeks after baseline, participants completed the 3 measures a final time, and those in the treatment group also filled out the feedback questions asking about their experiences with the app. Participants in both groups were presented with a debriefing statement that provided more information about the study. Those in the control group were given an opportunity to download and use the app at that time.

Email reminders about outstanding surveys were sent throughout the week as necessary to facilitate participant compliance. In addition, participants in the treatment group were sent a general reminder about the intervention once per week.

### Data Analysis

Quantitative data from the study questionnaires were entered into SPSS (version 26; IBM Corp) for statistical analysis. Comparisons between the treatment and control groups were performed using a series of mixed ANOVA statistical tests, with experimental group and health condition as the between-subjects factors; time point as the within-subjects factor; and scores on the depression, anxiety, and stress measures as dependent variables. Significant interactions were followed up with repeated measures ANOVAs and dependent *t* tests with Bonferroni adjustments. Baseline differences between the treatment and control groups were assessed using independent-sample *t* tests and chi-square tests of independence. All statistical tests were 2-tailed tests. The analysis of the quantitative data was blinded through the use of deidentification and dummy coding.

Qualitative data from the feedback questions were entered into NVivo (version 12; QSR International) and analyzed using inductive content analysis [39-41]. First, the lead researcher familiarized himself with the data by reading through the responses several times. Next, the responses were coded; new codes were created as needed when the researcher encountered data that did not fit an existing code. Once all responses had been coded, the researcher organized the codes into meaningful categories and subcategories. A second researcher coded the responses of 7 participants (approximately 20% of the treatment group data) and found high levels of agreement with the first researcher (Cohen κ=0.85). Results are presented in the final paper using narrative text and conceptual maps with frequency counts.

### Ethical Considerations

This study was approved by the research ethics board at the University of New Brunswick (036-2020). All participants provided informed consent at the outset of the study (details are included in the *Procedures* section). The study surveys were anonymous and linked over time through the use of a participant-generated identifier. The surveys included an embedded statement referring participants to their primary care provider or Crisis Services Canada if they experienced an urgent need for mental health treatment during the study. Participants were offered a CAD $20 (US $14.53) gift card to a retailer of their choice for completing the study. Participants who dropped out of the study before its completion were eligible for a CAD $5 (US $3.63) gift card.

## Results

### Participant Details

**Figure 1:**
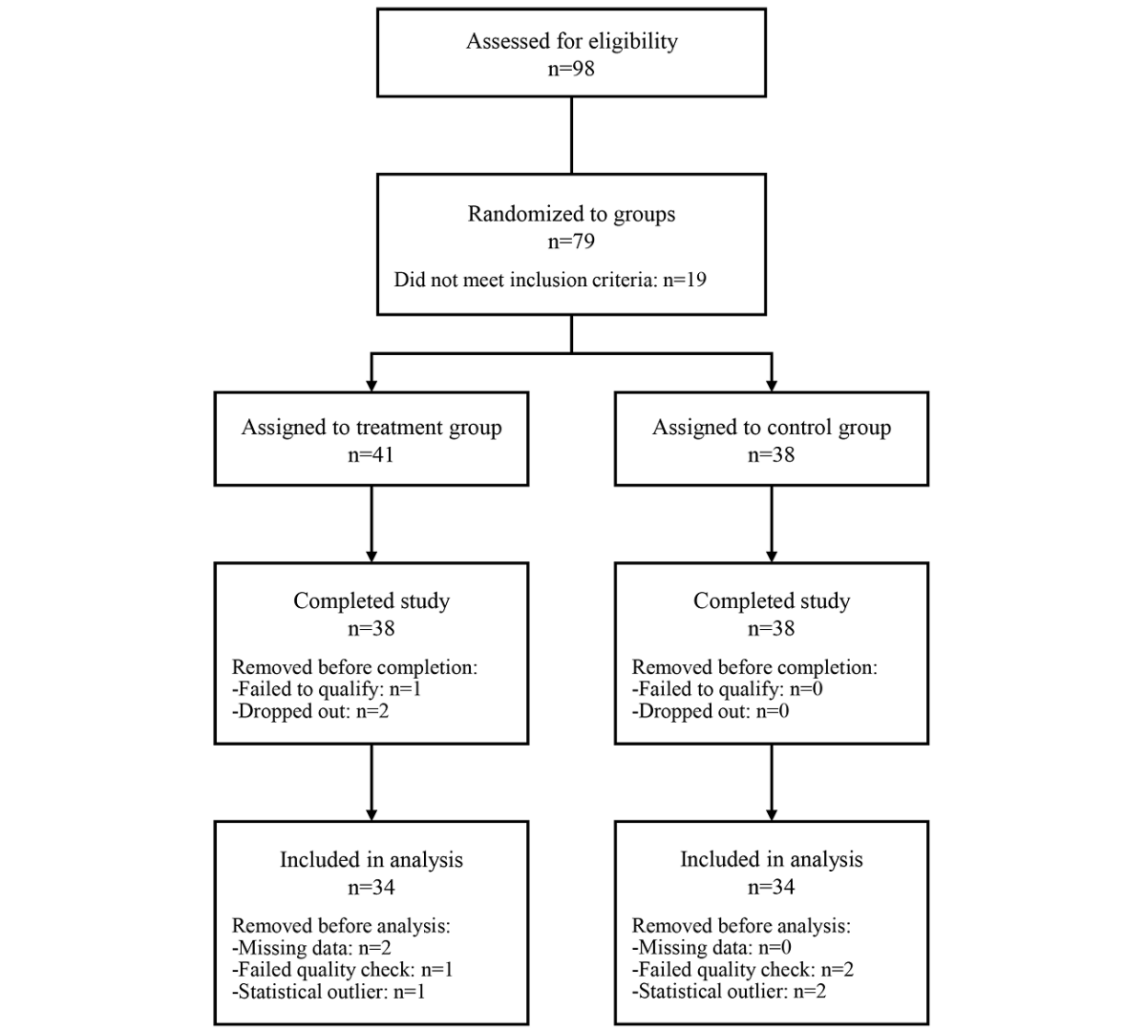
Participant flowchart.

A total of 98 individuals were assessed for eligibility, 19 of whom failed to meet the eligibility criteria. The remaining 79 individuals were randomized to the 2 experimental groups and started the study. One participant was removed from the study after the researchers discovered that he failed to meet the eligibility criteria, despite his claims upon recruitment. Two participants dropped out of the study, both within the first week: 1 participant had trouble installing the Wysa app, and another participant disliked talking to the chatbot. Finally, 8 participants were excluded during the data screening process for missing data, failing quality check questions embedded in the questionnaires, or providing outlying scores on one or more measures. Figure 1 illustrates participant flow in more detail.

After accounting for withdrawals and exclusions, we were left with a final sample of 68 participants. The sample consisted of 47 (69%) women, 20 (29%) men, and 1 (1%) transgender man. The mean age for the sample was 42.87 (SD 11.27) years, and participants were primarily White (n=50, 74%) and Asian (n=9, 13%). There were 36 (53%) participants with arthritis and 32 (47%) participants with diabetes. Most of the 36 participants with arthritis had a single form of arthritis: 14 (39%) had rheumatoid arthritis, 12 (33%) had osteoarthritis, and 7 (19%) had another type of arthritis. There were also 3 (8%) participants with multiple forms of arthritis. Among the 32 participants with diabetes, 25 (78%) had type 2 diabetes and 7 (22%) had type 1 diabetes. Participants with arthritis and diabetes were split evenly between the treatment and control groups, such that there were 18 people with arthritis and 16 people with diabetes in each group. No differences between the treatment and control groups were found in terms of demographic variables or baseline scores on the depression, anxiety, or stress measures (all *P* values >.05).

### Quantitative Analysis

#### PHQ-9 (Depression)

The mean PHQ-9 score at baseline was 8.24 (SD 6.13), which corresponds to mild depression. Results indicate that the severity of participants’ depression changed over the 3 time points of the study (*F*_2,128_=10.26, *P*<.001, η_p_^2^=.14), although this main effect was qualified by an interaction between experimental group and time point (*F*_2,128_=7.92, *P*<.001, η_p_^2^=.11). While the treatment group reported a decrease in depression severity over the study period (*F*_2,66_=16.73, *P*<.001, η_p_^2^=.34), no change was found in the control group (*F*_2,66_=0.32, *P*=.73, η_p_^2^=.01). With respect to the treatment group, there was a decrease in depression severity from baseline to week 2 (*P*=.04), with a further decrease from week 2 to week 4 (*P*=.001); the difference between baseline and week 4 was also significant (*P*<.001). Table 1 shows the means and SDs.

Participants with arthritis reported higher levels of depression severity than participants with diabetes over the study period (*F*_1,64_=8.73, *P*=.004, η_p_^2^=.12). However, no interaction was found for health condition and time point (*F*_2,128_=0.62, *P*=.54, η_p_^2^=.01) or for health condition, experimental group, and time point (*F*_2,128_=0.05, *P*=.95, η_p_^2^=.00). These results suggest that within-subject outcomes were relatively similar across both health conditions. In the treatment group, participants with diabetes dropped from mild depression at baseline (mean 6.75, SD 5.85) to minimal depression at week 4 (mean 3.75, SD 2.72); participants with arthritis dropped from moderate depression at baseline (mean 10.33, SD 5.83) to mild depression at week 4 (mean 6.61, SD 4.72). In the control group, participants with diabetes reported mild depression at baseline (mean 5.69, SD 4.92) and week 4 (mean 5.75, SD 5.42), whereas participants with arthritis reported mild-to-moderate depression at baseline (mean 9.72, SD 6.88) and week 4 (mean 9.17, SD 6.78).

**Table 1:** Means and SDs of depression, anxiety, and stress measures, by experimental group and time point.

Measures			Baseline, mean (SD)		Week 2, mean (SD)		Week 4, mean (SD)
**PHQ-9^a^ (depression)**							
	Treatment group	8.65 (6.03)		7.18 (4.90)		5.26 (4.11)	
	Control group	7.82 (6.29)		7.41 (5.88)		7.56 (6.33)	
**GAD-7^b^ (anxiety)**							
	Treatment group	7.44 (5.37)		5.85 (4.02)		4.74 (3.30)	
	Control group	6.09 (6.27)		6.32 (6.66)		6.56 (6.45)	
**PSS-10^c^ (stress)**							
	Treatment group	19.00 (6.79)		18.56 (6.57)		17.06 (6.21)	
	Control group	17.53 (8.83)		17.47 (7.82)		17.35 (7.93)	

^a^PHQ-9: Patient Health Questionnaire–9.

^b^GAD-7: Generalized Anxiety Disorder Scale–7.

^c^PSS-10: Perceived Stress Scale–10.

#### GAD-7 (Anxiety)

The mean GAD-7 score at baseline was 6.76 (SD 5.83), which corresponds to mild anxiety. Results indicate that the severity of participants’ anxiety changed over the 3 time points of the study (*F*_2,128_=4.28, *P*=.02, η_p_^2^=.06), although this main effect was qualified by an interaction between experimental group and time point (*F*_2,128_=8.15, *P*<.001, η_p_^2^=.11). While the treatment group reported a decrease in depression severity over the study period (*F*_2,66_=11.76, *P*<.001, η_p_^2^=.26), no change was found in the control group (*F*_2,66_=0.40, *P*=.67, η_p_^2^=.01). With respect to the treatment group, there was a decrease in anxiety severity from baseline to week 2 (*P*=.050), with a further decrease from week 2 to week 4 (*P*=.02); the difference between baseline and week 4 was also significant (*P*<.001). Table 1 shows the means and SDs.

Participants with arthritis reported higher levels of anxiety severity than participants with diabetes over the study period (*F*_1,64_=9.06, *P*=.004, η_p_^2^=.12). However, no interaction was found for health condition and time point (*F*_2,128_=0.56, *P*=.57, η_p_^2^=.01) or for health condition, experimental group, and time point (*F*_2,128_=0.79, *P*=.45, η_p_^2^=.01). Once again, these results suggest that within-subject outcomes were similar across both health conditions. In the treatment group, participants with diabetes dropped from mild anxiety at baseline (mean 5.38, SD 5.04) to minimal anxiety at week 4 (mean 3.06, SD 2.05); participants with arthritis dropped from mild-to-moderate anxiety at baseline (mean 9.28, SD 5.09) to mild anxiety at week 4 (mean 6.22, SD 3.52). In the control group, participants with diabetes reported minimal-to-mild anxiety at baseline (mean 4.69, SD 5.91) and week 4 (mean 4.56, SD 5.11), whereas participants with arthritis reported mild anxiety at baseline (mean 7.33, SD 6.49) and week 4 (mean 8.33, SD 7.12).

#### PSS-10 (Stress)

The mean PSS-10 score at baseline was 18.26 (SD 7.85), which corresponds to moderate stress. No significant changes in participants’ stress levels were found over the 3 time points of the study (*F*_2,128_=2.22, *P*=.11, η_p_^2^=.03). No interactions were found between experimental group and time point (*F*_2,128_=1.87, *P*=.16, η_p_^2^=.03); health condition and time point (*F*_2,128_=0.74, *P*=.48, η_p_^2^=.01); or health condition, experimental group, and time point (*F*_2,128_=1.81, *P*=.17, η_p_^2^=.03). However, participants with arthritis did report higher levels of stress than participants with diabetes over the study period (*F*_1,64_=6.37, *P*=.01, η_p_^2^=.09). Participants with diabetes reported moderate stress at baseline (mean 15.75, SD 8.29) and week 4 (mean 15.34, SD 6.45). Participants with arthritis also reported moderate stress at baseline (mean 20.50, SD 6.8) and week 4 (mean 18.86, SD 7.27), although their scores were generally higher than those reported by participants with diabetes.

### Qualitative Analysis

#### Feedback: Participant Likes

**Figure 2:**
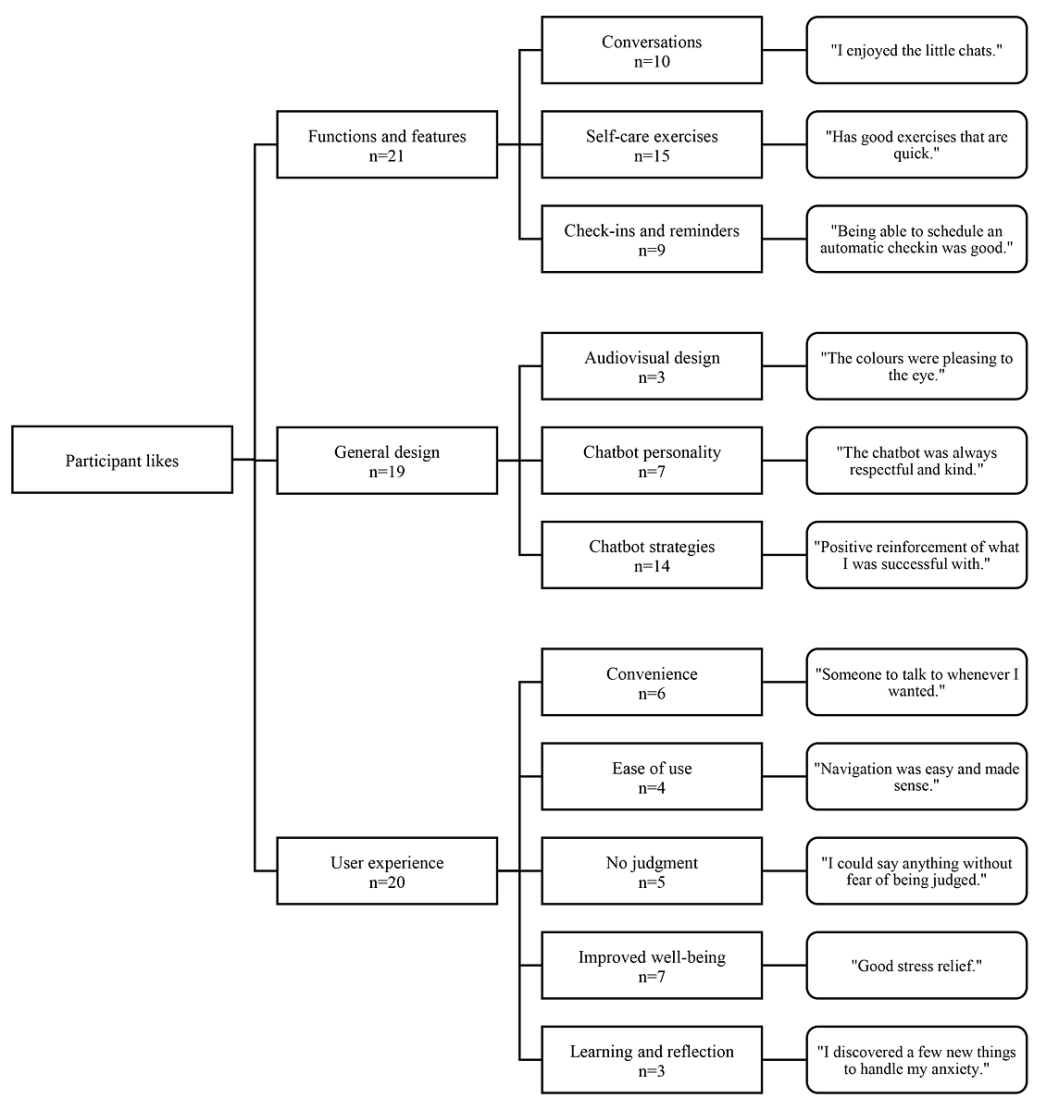
Conceptual map of participant likes.

Participants mentioned several things that they liked about their interactions with the Wysa app. Their responses fell into 3 major categories: functions and features, general design, and user experience. Each of these categories had its own subcategories, which are described in more detail in the following paragraphs. Figure 2 shows a conceptual map of the categories and subcategories. Supporting quotes are provided in the conceptual map.

In the functions and features category, 3 subcategories were identified. Participants said that they enjoyed conversations with the chatbot, describing them as “interesting” and “intuitive.” They also said that they enjoyed the various self-care exercises that were available within the app, such as sleep exercises, mindfulness exercises, and meditation. Finally, participants stated that they liked the check-in and reminder functionality, whereby the chatbot would check in with the user at specified times and provide reminders about various topics or activities.

In the general design category, 3 subcategories were identified. Participants said that they liked the audiovisual design of the app, particularly the colors, the font, and the voice that accompanied certain exercises and activities. They also liked the personality of the chatbot, describing it as “kind” and “reassuring,” among other qualities. Finally, participants liked the various strategies that were used by the chatbot, such as providing positive reinforcement and offering alternate perspectives.

In the user experience category, 5 subcategories were identified. Participants said that they found the app convenient and accessible, and they thought that it was easy to use as well. They said that they felt safe and unjudged when they were interacting with the chatbot, as the chatbot was not evaluating them as a person might. They also felt that the chatbot improved their well-being, largely by reducing stress and providing companionship. Finally, some participants mentioned that they were able to experience learning and reflection through the app.

#### Feedback: Participant Dislikes

**Figure 3:**
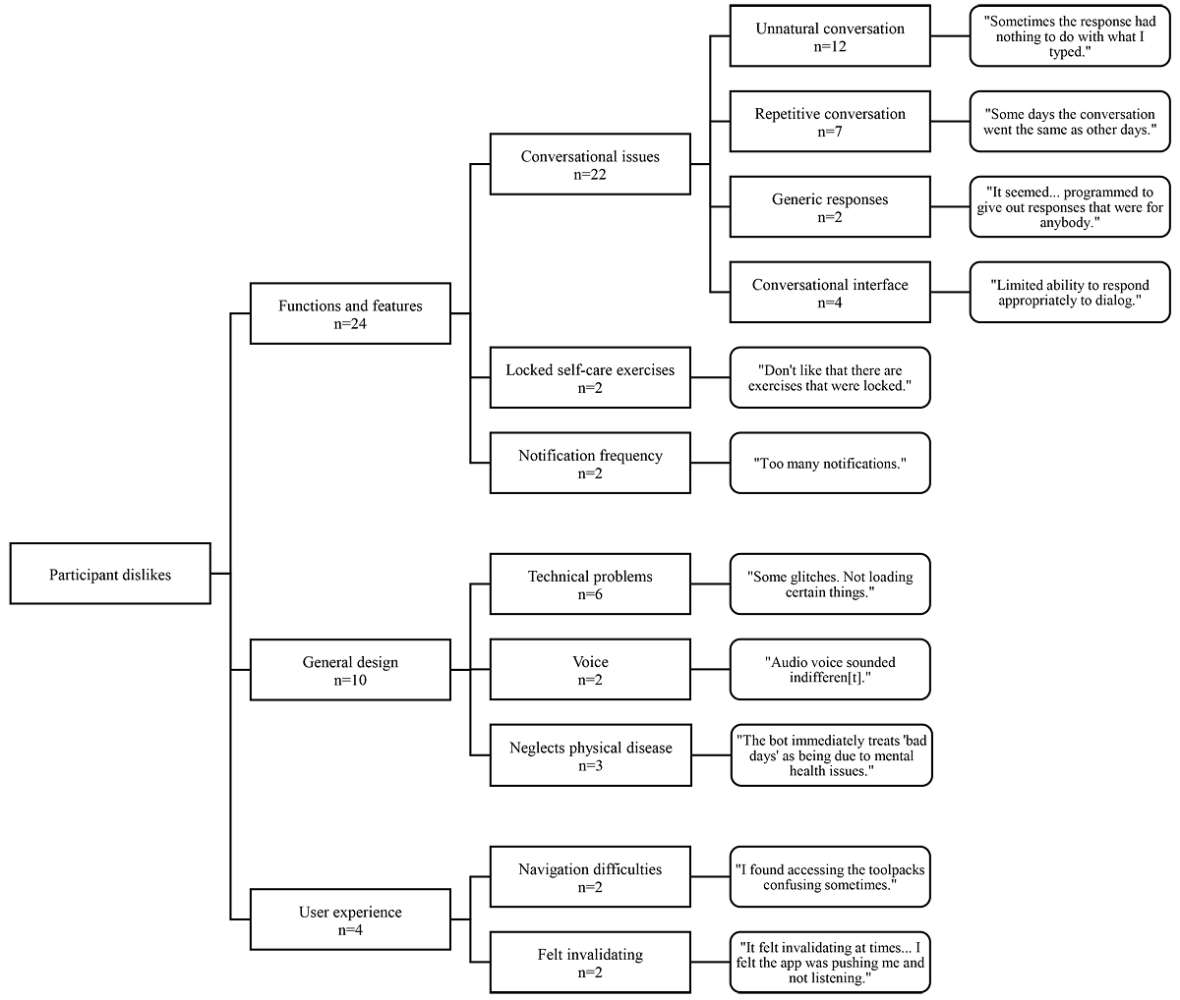
Conceptual map of participant dislikes.

Participants mentioned several things that they disliked about their interactions with the Wysa app. Their responses fell into the same 3 categories that were identified with participant likes: functions and features, general design, and user experience. Each of these categories had its own subcategories, which are described in more detail in the following paragraphs. Figure 3 shows a conceptual map of the categories and subcategories. Once again, supporting quotes are provided in the conceptual map. It is worth noting that most dislikes centered on 1 aspect of the app in particular, namely the chatbot’s conversational abilities.

In the functions and features category, 3 primary subcategories were identified. Despite enjoying conversations with the chatbot, participants stated that there were several issues with these conversations. More specifically, they said that conversations were sometimes unnatural (ie, there was a disconnect between user input and chatbot output) and repetitive, and that the chatbot’s responses were generic or not individualized. They also disliked certain aspects of the conversational interface, such as having limited response options when replying to particular questions. Beyond these conversational issues, some participants disliked the fact that several self-care exercises were locked when first using the app. Some participants also disliked the frequency of notifications, although it should be mentioned that notification frequency can be adjusted within the app.

In the general design category, 3 subcategories were identified. Participants encountered occasional technical problems with the app, such as freezing or a failure to load. Some participants also disliked the voice that was used within the app, finding it indifferent or unpleasant. In addition, a few participants were disappointed that the app did not have more content related to physical disease or better recognize that their “bad days” may be due to physical symptoms versus mental health issues.

In the user experience category, 2 subcategories were identified. Although several participants said that they found the app easy to use, some participants did have difficulty navigating the app. In addition, 2 participants felt that interacting with the chatbot was “invalidating” and that they were not being heard or respected.

## Discussion

### Principal Findings

The results of this study suggest that mental health chatbots can be an effective source of mental health support for people with chronic diseases, specifically individuals with arthritis or diabetes. Participants in the treatment group reported decreases in depression and anxiety severity after using a mental health chatbot over a 4-week period. These decreases were evident by the 2-week mark, and further reductions were seen over the final 2 weeks of the study period. By comparison, no significant changes in depression or anxiety severity were apparent in the control group. No changes in participants’ stress levels were found in either experimental group, although there was some descriptive evidence in the participant feedback that the chatbot app reduced stress for some individuals.

Participants with arthritis reported higher levels of depression and anxiety severity than participants with diabetes over the course of the study, as well as higher levels of stress. However, the general pattern of results was similar across these health conditions. The findings on depression and anxiety are of particular interest due to the impact of the intervention on these outcomes. On average, participants with arthritis reported mild depression and anxiety after using the app for 4 weeks, whereas participants with diabetes reported minimal depression and anxiety. These levels were improvements over the baseline mental health states reported for these conditions. No changes in depression or anxiety severity were evident in the control group, regardless of health condition.

Responses to the feedback questions offer insight into participants’ experiences with the chatbot and the Wysa app more generally. Participants said that they liked the functions and features of the app, the general design of the app, and the user experience. Their comments on the user experience were particularly insightful; they said that they found the app convenient and easy to use, appreciated the lack of judgement from the chatbot, and experienced improved well-being and some degree of learning and reflection thanks to the program. Participants’ comments on what they disliked about the app were also informative. Although participants touched on several points in their feedback, most of their dislikes centered on conversational issues. Participants tended to enjoy conversations with the chatbot in a general sense, but they said that these conversations were unnatural and repetitive at times, and that the chatbot’s responses were too generic. They also disliked certain aspects of the conversational interface.

### Comparisons to Previous Work

Past research has shown that mental health chatbots can reduce depression and anxiety severity across a variety of populations [19-21,23,24]. Our study provides further support for the effectiveness of this technology, highlighting its ability to address depression and anxiety in certain chronic disease populations. Some of the previous studies in this area used a similar assessment period as our study, which allows for a direct comparison of the depression and anxiety results. For example, decreases in depression and anxiety severity in our treatment group were greater than those reported by Fulmer et al [20], who asked university students to use a mental health chatbot over a 4-week time frame. Meanwhile, the decreases in our treatment group were somewhat less than those reported by Daley et al [18], who asked a general population sample to use a mental health chatbot over a 30-day period. However, participants in this latter study reported higher levels of depression and anxiety severity at baseline than our own participants, suggesting that there was a greater opportunity for improvement in that sample.

Although some research has indicated that mental health chatbots can decrease stress in nonclinical populations [18,22], no significant reductions in stress were found in our chronic disease sample, at least in terms of the quantitative data. These divergent results might be attributed to differences in the study populations or the content and functionality of the chatbots that were tested in these studies. In addition, the measure that was used to assess participant stress in our study could have contributed to the lack of significant results. The PSS-10 assesses stress over the previous month, a longer time frame than the period covered by the depression and anxiety measures. Given that participants were only enrolled in the study for 4 weeks, it is possible that stress experienced just before or early in the study was factoring into the ratings of stress reported at the final assessment point.

Participants’ feedback on the intervention was generally consistent with past findings from the literature on mental health chatbots. For instance, previous research has highlighted the convenience of these programs [20,22], their ability to stimulate learning and reflection [19,22], and their perceived safeness and lack of judgment [42,43], all points that were discussed by participants in our study. The convergence of our results with past findings suggests that there are probably global benefits to mental health chatbots that transcend specific programs. Participants’ dislikes also aligned with past research, particularly with respect to conversational issues. Participants in previous studies have described conversation with mental health chatbots as somewhat unnatural, repetitive, and shallow [19,20,22], sentiments that were echoed in our sample. This feedback indicates that the simulation of genuine human conversation is an ongoing challenge for the developers of these programs, and that improving conversational abilities should be a key goal for developers in the coming years.

### Practical Implications

Although mental health support could be beneficial for many people with chronic diseases, these individuals face barriers to accessing conventional forms of mental health treatment [13-17]. The results of this study indicate that mental health chatbots may be an effective source of support for people with these health conditions. These programs are not a replacement for real mental health professionals [44-46], and their limitations, particularly with respect to their conversational abilities, suggest that they are probably not appropriate for serious cases [46-48]. Moreover, not everyone likes or wants to use these programs. Consistent with this idea, 1 participant in this study dropped out because she disliked talking to the chatbot, and 2 participants who completed the study felt that interacting with the chatbot was invalidating and that they were not being heard or respected. Regardless, mental health chatbots could be an accessible and cost-effective resource for those individuals who have less serious mental health issues and are open to automated technology-based solutions.

### Limitations and Future Directions

This study has several limitations. The control group that was used for the study was a no-treatment control group that received no intervention or manipulation from the researchers. This specific type of control group represents an untreated population whose mental health may naturally improve, decline, or show no changes over time. A no-treatment control group is useful for establishing the presence of an effect in the treatment group [49]. However, it does not allow researchers to evaluate the likelihood or extent of a placebo effect, nor does it show how the treatment under investigation compares to standard treatments in this area (eg, support from real mental health professionals). In future studies, researchers should supplement a no-treatment control group with a placebo group or an active control group to better understand the scope of any effects.

The generalizability of the results is also unclear. Due to the technical requirements of the chatbot app, participation in this study was limited to individuals who had access to a smartphone with an active internet connection. It is possible that these individuals are more comfortable with technology than people without connected smartphones (or smartphones more generally), in which case they may be more receptive and responsive to mental health chatbots. Participation was also limited to individuals between the ages of 19 and 65 years, as it was thought that this age group would be most likely to have access to smartphones and the digital skills required to use them. It is unclear whether the study results would apply to older or younger age groups. In future studies, researchers may want to include other age groups and offer their chatbots through a variety of delivery channels so that they capture a broader cross-section of individuals.

Limitations surrounding the selected health conditions also warrant some comment. Arthritis and diabetes are not homogenous diseases, and it is possible that patterns of results might have varied across subtypes of these conditions (eg, type 1 vs type 2 diabetes). In the future, researchers working with arthritis or diabetes populations may want to target a larger sample size so that these subtypes can be appropriately compared. Studies addressing other chronic diseases should also be carried out. This study focused on arthritis and diabetes due to the prevalence of these conditions and the broad body of literature linking them to mental health issues. It is unclear how well the results of this study would translate to other chronic diseases, especially those with a particularly poor prognosis (eg, certain types of cancer). Similarly, it is unclear whether the presence of comorbid health conditions has an impact on chatbot effectiveness. Researchers may want to examine these issues in future studies to gain a better understanding of the usefulness of this technology.

### Conclusion

The results of this study suggest that mental health chatbots can be an effective source of mental health support for people with chronic diseases such as arthritis and diabetes. These programs are not intended to replace real mental health professionals, and they are not well suited for all individuals. However, they are an accessible and cost-effective resource that could benefit people with chronic diseases who have less serious mental health issues and are open to technological solutions.

## Appendix

Multimedia Appendix 1 CONSORT-eHEALTH checklist (V 1.6.1).

## Abbreviations

CONSORT: Consolidated Standards of Reporting Trials
GAD-7: Generalized Anxiety Disorder Scale–7
PHQ-9: Patient Health Questionnaire–9
PSS-10: Perceived Stress Scale–10

## Data Availability

The data set generated during and/or analyzed in this study is available on Open Science Framework [50].
